# New Trends in Injection-Based Therapy for Thumb-Base Osteoarthritis: Where Are We and where Are We Going?

**DOI:** 10.3389/fphar.2021.637904

**Published:** 2021-04-13

**Authors:** Sara Tenti, Sara Cheleschi, Nicola Mondanelli, Stefano Giannotti, Antonella Fioravanti

**Affiliations:** ^1^ Department of Medicine, Surgery and Neuroscience, Rheumatology Unit, Clinic for the Diagnosis and Management of Hand Osteoarthritis, Azienda Ospedaliera Universitaria Senese, Siena, Italy; ^2^ Department of Medicine, Surgery and Neuroscience, Orthopedics and Traumatology Unit, University of Siena, Siena, Italy

**Keywords:** thumb-base osteoarthritis, trapezio-metacarpal osteoarthritis, first carpo-metacarpal osteoarthritis, rizoartrhosis, intra-articular injection, hyaluronic acid, corticosteroids, platelet-rich plasma

## Abstract

Thumb-base osteoarthritis (TBOA) is a common condition, mostly affecting post-menopausal women, often inducing a significant impact on quality of life and hand functionality. Despite its high prevalence and disability, the therapeutic options in TBOA are still limited and few have been investigated. Among the pharmacological strategies for TBOA management, it would be worthwhile to mention the injection-based therapy. Unfortunately, its efficacy is still the subject of debate. Indeed, the 2018 update of the European League Against Rheumatism (EULAR) recommendations for the management of hand osteoarthritis (OA) stated that intra-articular (IA) injections of glucocorticoids should not generally be used, but may be considered in patients with painful interphalangeal joints, without any specific mention to the TBOA localization and to other widely used injections agents, such as hyaluronic acid (HA) and platelet-rich plasma (PRP). Even American College of Rheumatology (ACR) experts conditionally recommended against IA HA injections in patients with TBOA, while they conditionally encouraged IA glucocorticoids. However, the recommendations from international scientific societies don’t often reflect the clinical practice of physicians who routinely take care of TBOA patients; indeed, corticosteroid injections are a mainstay of therapy in OA, especially for patients with pain refractory to oral treatments and HA is considered as a safe and effective treatment. The discrepancy with the literature data is due to the great heterogeneity of the clinical trials published in this field: indeed, the studies differ for methodology and protocol design, outcome measures, treatment (different formulations of HA, steroids, PRP, and schedules) and times of follow-up. For these reasons, the current review will provide deep insight into the injection-based therapy for TBOA, with particular attention to the different employed agents, the variety of the schedule treatments, the most common injection techniques, and the obtained results in terms of efficacy and safety. In depth, we will discuss the available literature on corticosteroids and HA injections for TBOA and the emerging role of PRP and other injection agents for this condition. We will consider in our analysis not only randomized controlled trials (RCTs) but also recent pilot or retrospective studies trying to step forward to identify satisfactory management strategies for TBOA.

## Introduction

Thumb-base osteoarthritis (TBOA) is a highly prevalent condition affecting middle-aged and older people; the condition increases with age, is more common in women—particularly post-menopausal—and it is often bilateral (Dahaghin et al., 2005; Haugen et al., 2011; Kloppenburg et al., 2017).

The prevalence of symptomatic TBOA among people aged >50 years was estimated from 5 to 7%, while the prevalence of radiographic TBOA is higher, ranging from 45 to 60% (Sodha et al., 2005; Sonne-Holm and Jacobsen, 2006).

The main symptoms of TBOA are pain, localized to the base of the thumb, stiffness, tenderness and loss of range of motion. The impairment function reduces the ability to perform activities of daily living, such as writing, opening a jar, turning a car key, and turning a door or handling small objects. In the more advanced stages, thenar muscle wasting combined with subluxation and adduction of the thumb metacarpal can induce a characteristic “squaring” joint deformity. Furthermore, patients with concomitant osteoarthritis (OA) of the interphalangeal (IP) joints and TBOA complain of more pain, functional disability, and reduced quality of life (Bijsterbosch et al., 2010; Tenti et al., 2020).

Despite its high prevalence and disability, the therapeutic options for TBOA are still limited and few investigated; its management usually requires a combination of non-pharmacological, pharmacological, and surgical strategies with a multidisciplinary approach (Kloppenburg et al., 2017).

Among the pharmacological strategies, it would be worthwhile to mention the use of intra-articular (IA) injection-based therapy with corticosteroid or hyaluronic acid (HA). Unfortunately, its efficacy is still the subject of debate and not universally shared by the current guidelines for the management of hand OA.

The 2007 European League Against Rheumatism (EULAR) recommendations for hand OA support the use of IA long-acting corticosteroids for painful flares of OA, especially for TBOA (Zhang et al., 2007).

Conversely, the 2018 update of EULAR recommendations state that IA injections of steroids should not generally be used, but may be considered in patients with painful IP joints, without any specific mention to the TBOA localization and to other widely used IA agents, as HA and platelet-rich plasma (PRP) (Kloppenburg et al., 2019). Even American College of Rheumatology (ACR) experts conditionally recommend against IA HA injections in patients with TBOA, while they conditionally encourage IA glucocorticoids (Kolasinski et al., 2020).

However, the recommendations from international scientific societies do not often reflect the clinical practice of all physicians who routinely take care of TBOA patients; indeed, corticosteroid injections are a mainstay of therapy in OA, especially for patients with pain refractory to oral treatments, and HA is considered as a safe and effective therapeutic option.

Considering the high prevalence of a disabling disease, such as TBOA, we aimed to perform a narrative review analyzing the current evidence on the efficacy and safety of the intra-articular therapy. For this purpose, we grouped the literature evidence for different used IA drugs (corticosteroids, hyaluronate, PRP, or other medications), adding a discussion to find the gaps in this area and to identify where additional research is needed.

## Methods

### Data Sources and Searches

We created a comprehensive search strategy aimed to capture all relevant papers concerning injection-based therapy for TBOA. The search strategy was applied to the following bibliographic databases: Cochrane Library, PubMed, MEDLINE, EMBASE, Web of Science, and Scopus, using the terms “thumb-base joint osteoarthritis,” “trapezio-metacarpal joint osteoarthritis,” “first carpo-metacarpal joint osteoarthritis,” “rizoartrhosis” in combination with “intra-articular injections,” “injection-based therapy,” “steroid injections,” “hyaluronic acid injections,” “platelet-rich plasma injections,” and “prolotherapy.” Additional articles were identified by searching bibliographies of each paper. Furthermore, we searched www.clinicaltrials.gov for active and/or recently completed clinical trials testing agents for IA therapy of TBOA.

We conducted the search of the literature in October 2020.

### Inclusion/Exclusion Criteria

In this narrative review, we included all studies analyzing an injection-based intervention for patients suffering from TBOA. In particular, articles were considered eligible if they met the following criteria: 1) diagnosis of TBOA of the study population, according to the ACR criteria for hand OA (Altman et al., 1990); 2) any study design, including not only randomized controlled trials (RCTs), but even prospective open label or retrospective studies; 3) any studies presenting at least an evaluation of the efficacy, in terms of both pain and function, and tolerability of injection-based therapy; 4) any type of pharmacological agents or medical devices injected; 5) any injection approach included (with any or no image guidance); 6) studies published from 2000 to October 2020, totally written in English language. Studies were excluded if they did not evaluate the effects of injection therapy on both pain and function; review articles, studies not published as a full article (conference abstracts) and papers not totally written in the English language were also not considered.

### Selection of Studies

Initially, duplicates were removed and relevant trials were independently screened by checking titles, keywords, and abstracts by two authors (T. S., M. N.). The references of the selected articles and all significant reviews on the topic were also checked to identify other potential papers. Then, a full-text evaluation of the selected studies was performed by the same authors (T. S, M. N.) to determine whether the trials met the inclusion criteria regarding design, study population, outcomes, and interventions. Disagreement between the two reviewers was solved by involving a third author (F. A.).

### Data Extraction

Data were independently extracted and aggregated into a Microsoft Excel®spreadsheet database by two authors (C. S. and G. S.). In particular, the data extraction sheet was designed to collect data about the study design, participants, details on the interventions undertaken, types of outcome measures evaluated, duration of follow-up, loss to follow-up, and results. Any inconsistencies between the two authors were solved by consensus discussion or by involving a third reviewer (F. A.) in case of persistent disagreement.

### Outcomes and Data Analysis

Patient-reported pain and function were considered the main outcomes of interest; possible side effects related to the injection-based therapy were also recorded. A priori we defined as short-term follow-up, a follow-up period ranging from one week to 3 months, medium-term follow-up a period ranging from 3 to 6 months, and long-term follow-up above 6 months. Descriptive analysis was performed for all demographic data, interventions, and outcome parameters to facilitate narrative interpretation and comparison among the studies.

## Results

### Literature Search Results and Trials Characteristics

**FIGURE 1:**
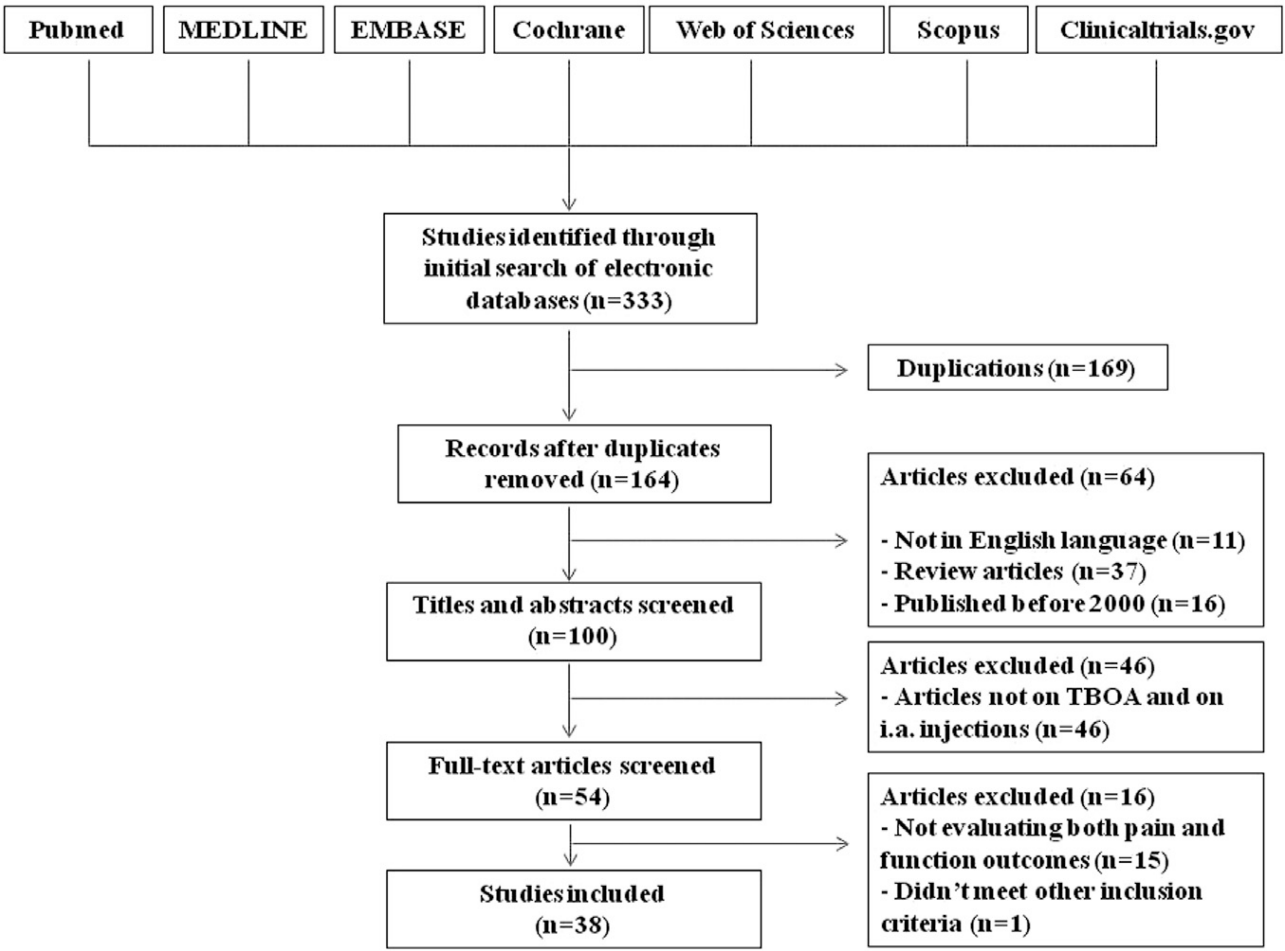
Study flow diagram. TBOA, thumb-base osteoarthritis.

In total, 164 potential eligible studies were found; no additional papers were obtained by hand searching of references. Of these, 11 studies were excluded because they were written in a language other than English, 37 because they were review articles and 16 because were published before 2000. Based on the title and the abstract content, 46 of these articles were not included in our review. The full texts of the remaining 54 studies were read, and a further 16 studies were excluded because they did not meet other inclusion criteria (Figure 1). We identified 38 assessable studies, six analyzing the effect of IA injections of corticosteroids, 20 evaluating the effects of IA hyaluronic acid, of whom seven in comparison to steroids, five dealing with IA injections of PRP and the remaining seven exploring new emerging IA therapy. Additionally, we identified two study protocols for trials planned for the coming years.

### Corticosteroid Injections

Intra-articular corticosteroids have been used for decades in the management of symptomatic OA and remain a common practice given their potent anti-inflammatory properties and the favorable cost/effectiveness profile. Steroid injection is typically reserved to patients not responding to systemically delivered drugs or who do not tolerate pharmacological treatments (Jüni et al., 2015). The choice of the drug depends on the experience and preference of the physician, but generally includes triamcinolone, methylprednisolone, and betamethasone.

Intra-articular injection of steroid is mostly used and studied for inflammatory and degenerative disease of large joints, such as the knee, while the scientific evidence for TBOA is limited and conflicting. The characteristics of the few studies found by our literature research are summarized in Table 1.

**TABLE 1:** Summary of studies investigating intra-articular injections of corticosteroids for the treatment of thumb-base osteoarthritis.

Authors, publication year	Study design	Sample size (pts)	Inclusion criteria^a^	Intervention	Follow-up duration (weeks)	Injection guidance	Outcomes evaluated	Results
Jahangiri et al. (2014)	Double-blind RCT	60	-Age >40 years	Group I: 2 monthly injections of 0.9% saline/1 ml followed by methylprednisolone acetate 40 mg/0.5 ml mixed with 2% lidocaine/0.5 ml after 1 month	24	None	VAS pain (0–100); tenderness intensity; HAQ-DI (0–3); pinch grip strength (lb)	The results on pain were better for steroid group at 1 month, and for dextrose group at 6 months; more effectiveness on functionality measures was observed for dextrose after 6 months
			-Duration of pain ≥3 months					
			-Pain intensity >30/100 mm VAS	Group II: 3 monthly injections of 20% dextrose/0.5 ml mixed with 2%lidocaine/0.5 ml				
			-Eaton grade >1					
NCT00685880	Double-blind RCT	2	-Age > 45 years	Group I: One injection of 10% dextrose solution	24	None	VAS pain (0–10); analgesic use; grip strength; functional assessment of upper extremities	Early termination due to low enrollment; no subject data was analyzed
			-Eaton grade 2–3					
			-Pain >3/10 on VAS	Group II: one injection of betamethasone 3 mg/0.25–0.5 ml (CELESTONE® SOLUSPAN®)				
			-Symptoms duration >6 months					
Day et al. (2004)	Open prospective study	30	-Isolated pain at TB	One injection of methylprednisolone acetate 40 mg/1 ml, mixed with 1% lidocaine/0.5 ml, 0.5% bupivacaine hydrochloride/0.5 ml and bicarbonate 0.5 ml followed by immobilization in a thumb spica splint for 3 weeks	72	None	Subjective pain relief (0–10); DASH (0–100)	Steroid injection with splinting provided long-term (until 18 months) benefit in early stage of the disease (eaton stage 1)
			-Tenderness over the TMCJ					
			-Positive grind test					
Joshi (2005)	Open prospective study	25	NR	One injection of methylprednisolone acetate 10 mg/0.25 ml	48	None	VAS pain (0–10); HAQ (0–3)	A significant long-term benefit wasn’t observed; only a significant improvement of pain was reported after 1 month
Khan et al. (2009)	Open prospective study	40	Not reported	One injection of kenalog 10 mg/0.5 ml and a local anesthetic solution (not better specified)	24	None	VAS pain (0–10); DASH (0–100)	All patients reported a significant improvement in pain and hand function (*p* < 0.05), regardless of the disease stage. Additionally, a marked difference in the duration of improvement in hand function between early and late stages of the disease (*p* = 0.0046) was described
Rocchi et al. (2018)	Prospective comparative study	50	-Eaton stage 1–2	Group I: One injection of methylprednisolone acetate 40 mg/1 ml and lidocaine 10 mg	48	None	TMC pain and restriction of activities (degrees); DASH (0–100); treatment satisfaction (1–10 scale); pinch strength (kg)	Group I reported a rapid decrease of pain and an increase of the functional performances, but this beneficial effect was short-lived. Group II experienced a more gradual improvement that lasted longer
			-Isolated pain at TB and tenderness over the TMCJ	Group II: 10 physical therapy sessions (including both physical agent application both exercise) with a hand therapist for 5 days a week for two consecutive weeks				
			-Positive grind test					

^a^ All studies included patients with diagnosis of TBOA according to the ACR criteria (Altman et al., 1990).

DASH, disabilities of the arm and shoulder; HAQ, health assessment questionnaire; HAQ-DI, health assessment questionnaire disability index; NR, not reported; pts, patients; TB, thumb-base; TMC, trapezio-metacarpal; TMCJ, trapezio-metacarpal joint; RCT, randomized controlled trial; VAS, visual analogue scale.

A double-blind RCT compared the efficacy of IA steroids (methylprednisolone acetate 40 mg) with a 20% dextrose solution (prolotherapy treatment), both mixed with 0.5 ml of 2% lidocaine (Jahangiri et al., 2014). In this study, sixty patients with TBOA beyond stage one of the Eaton classification (Eaton and Glickel, 1987) were selected and randomly assigned to corticosteroids or prolotherapy. One group received two monthly placebo injections with a 0.9% saline solution and in the third month the steroid, the other one was treated with three monthly IA dextrose solution. The efficacy of the treatment was evaluated at 1, 2, and 6 months after the third injection. Methylprednisolone appeared more effective in the short-term, but at the sixth month the results showed a remarkable difference in favor of dextrose. No severe side effects were reported for prolotherapy.

Another randomized double-blind trial comparing IA 10% dextrose solution to betamethasone injection for the treatment of symptomatic TBOA was performed by the Mayo Hand Clinic (Clinicaltrials.gov, NCT00685880). The study started in 2008, but it appears to have been discontinued because of the small number of patients recruited.

A number of non-RCTs investigated the effectiveness and tolerability of IA steroid for TBOA. Thirty patients with TBOA were included in a long-term prospective open study and treated with a single injection of methylprednisolone acetate (40 mg) and 0.5 ml of 1% lidocaine followed by the use of a thumb spica splint for three weeks (Day et al., 2004). The clinical evaluation provided long-term (until 18 months) benefit in early stage of the disease, while in the severe form of disease (Eaton stage 4) the treatment appeared ineffective. On the contrary, Joshi R (Joshi, 2005) in a prospective case series of 25 patients treated with a single injection of 10 mg of methylprednisolone acetate showed a significant improvement of pain after 1 month, but not in the following observations at 3, 6, and 12 months. The Author did not report any information about the stage of the disease or about the concomitant use of other pharmacological or non-pharmacological treatments during the study period.

Khan et al. (Khan et al., 2009) conducted a prospective open study in 40 patients with TBOA to evaluate the improvement in pain and function of the hand after a single IA corticosteroid injection (triamcinolone acetonide 10 mg) and a local anesthetic solution. The symptomatic effect was evident in all patients in the short-term evaluation (2–4 weeks), but the duration of this benefit was different according to the stage of the disease.

Rocchi et al. (Rocchi et al., 2018) compared, prospectively, the effect of 10 sessions of physiotherapy to a single IA injection of methylprednisolone acetate (40 mg) and lidocaine in 50 patients with TBOA at early stages. The patients receiving IA therapy reported a rapid decrease of pain and an increase of the functional performances, but this beneficial effect was not maintained in the long-term follow-up (12 months). The group treated with physiotherapy (heat application, passive and active mobilization, massage, and stretching) experienced a more gradual improvement that lasted longer.

We did not report in this analysis the trials by Meenagh et al. (Meenagh et al., 2004) and by Swindells et al. (Swindells et al., 2010), because they evaluated only the effects of IA steroids on pain and not on functionality, as determined by our inclusion criteria.

### Hyaluronic Acid Injections

HA represents another well-known IA treatment for OA; its use is based on its ability to restore the rheological properties of the synovial fluid and thus to decrease pain and improve functionality. For these reasons, it can represent a valid and safe alternative to IA corticosteroids in OA patients not responding to non-steroidal anti-inflammatory drugs (NSAIDs) and analgesics. The role of viscosupplementation with HA is nowadays worldwide recognized for the treatment of knee OA, but its usefulness has been recently suggested also for other joints, such as hip, ankle, shoulder, temporomandibular joint, and thumb (Henrotin et al., 2015). However, as demonstrated in recent systematic reviews and meta-analysis, the scientific evidence on the efficacy of the IA therapy with HA in TBOA is still subject of debate, and often limited by the great heterogeneity of the trials performed in this field (Trellu et al., 2015; Kroon et al., 2016; Riley et al., 2019). The main sources of heterogeneity are represented by different HA formulations employed with variable injection schedules and IA techniques, different periods of follow-up and a great variety of assessed outcomes.

We identified a total of 20 papers, including nine RCTs, two retrospective comparative studies and nine open label trials evaluating the effects of the IA therapy with HA in TBOA patients. In the controlled studies, the comparator treatment was represented by IA corticosteroids (7 papers), IA saline solution (one paper) and extracorporeal shock wave therapy (ESWT) (one paper). The remaining controlled trials evaluated different schedules of IA HA in one case, and assessed a combination therapy with IA HA and IA ketorolac vs. IA HA alone in another one.

### Hyaluronic Acid Versus Corticosteroids Injections

The individual characteristics of each study (6 RCTs and one retrospective comparative study) are reported in Table 2. A direct comparison among these trials is not possible, considering the great heterogeneity of the studies for a variety of parameters. Four research papers evaluated, as HA formulation, sodium hyaluronate from different commercial brands, two studies analyzed hylan and another one considered a hybrid formulation of HA. As corticosteroid comparator, the Authors chose triamcinolone acetonide in four cases, although with different dosages, betamethasone disodium phosphate in two works and methylprednisolone in the remaining one. Injections courses ranged from a single injection to three weekly injections. The length of follow-up was of 6 months for all trials, except from one in which the follow-up lasted until 12 months. Image guidance was employed in only one study. The only outcome parameter evaluated in all studies was pain by a visual analogue scale (VAS). Functionality was assessed by a variety of different tests.

**TABLE 2:** Summary of studies investigating intra-articular injections of hyaluronic acid for the treatment of thumb-base osteoarthritis.

Authors, publication year	Study design	Sample size (pts)	Inclusion criteria^a^	Intervention	Follow-up duration (weeks)	Injection guidance	Outcomes evaluated	Main results on pain and function	N^o^ of reported adverse events
Stahl et al. (2005)	RCT	52	NR	Group I: One injection of methylprednisolone acetate (depomedrol®) 40 mg/1 ml	24	None	VAS pain (0–10); grip and pinch strength (kg); PPT	A reduction of pain was observed in both groups after 1 month. Grip strength improved significantly in both groups at 6 months; patients treated with HA showed an improvement of pinch strength and PPT at 3 months, too	0
				Group II: one injection of sodium hyaluronate (orthovisc®) 15 mg/1 ml					
Fuchs et al. (2006)	Single-blind RCT	56	-VAS pain ≥40 mm for at least 6 months	Group I: 3 weekly injections of 1% sodium hyaluronate 10 mg/1 ml (ostenil® mini), average MW 1.2 milion dalton	26	None	VAS pain (0–100); lateral pinch grip (kg); pulp pinch grip (kg); radial and palmar ab/adduction and opposition (degrees)	VAS pain improved in a more significantly manner in group II at 2–3 weeks and in group I at 26 weeks. At the end of follow-up, a superiority of HA was found for the improvement of lateral pinch strength, pulp pinch strength and for radial abduction/adduction and opposition	0
			-Good general condition and compliance	Group II: 3 weekly injections of triamcinolone acetonide (volon® A10) 10 mg/1 ml					
Heyworth et al. (2008)	Double-blind RCT	60	NR	Group I: 2 weekly injections of 1 ml of hylan G-F 20 (synvisc®)	26	None	VAS pain (0–10); DASH (0–100); ROM (degrees); grip and pinch strength (lbs)	There were no statistically significant differences among the three studied groups for most of the outcome measures at any of the follow-up time points	0
				Group II: one injection of 1 ml placebo of normal saline (0.9% sodium chloride), followed by one week by an injection of 1 ml of sodium betamethasone sodium phosphate–betamethasone acetate (celestone soluspan®)					
				Group III: 2 weekly injections of 1 ml placebo of normal saline (0.9% sodium chloride)					
Monfort et al. (2015)	Single- blind RCT	88	-Age ≥ 18 years	Group I: 3 weekly injections of 500–1,000 kDa HA (suplasyn®) 5 mg/0.5 cm^3^, MW 500–1,000 kDa	24	Yes, US-guidance	VAS pain (0–10); FIHOA (0–30); SF-36 PCS and MCS (0–100)	VAS and FIHOA significantly improved trough follow-up without significant differences between groups. A sub-analysis of patients with FIHOA ≥ 5 and VAS ≥ 3 at baseline showed a significantly major improvement of FIHOA score in the HA group vs steroid group at 12 and 24 weeks	Group I: 5
			-Clinical symptoms for at least 90 days requiring analgesics or NSAIDs treatment	Group II: 3 weekly injections of 0.5 cm^3^ of betamethasone disodium phosphate 1.5 mg and betamethasone acetate 1.5 mg					Group II: 5
Tenti et al. (2017)	Retrospective comparative study	100	-Age between 45 and 75 years	Group I: 2 injections performed 15 days apart of a 3.2% hybrid formulation of HA (sinovial H-L®) 16 mg + 16 mg/1 ml; combination of 1,100–1,400 kDa MW and 80–100 kDa MW	24	None	VAS pain (0–100); FIHOA (0–30); HAQ (0–3); duration of morning stiffness (minutes); SF-36 PCS and MCS (0–100)	Both therapies provided effective pain relief and functional improvement, but the benefits achieved were significantly superior in group I vs group II, after 1 month and persisted until 6 months. HA was also associated to a significant improvement of morning stiffness, HAQ and SF-36 PCS	Group I: 2
			-Clinical symptoms for at least 3 months	Group II: 2 injections performed 15 days apart of triamcinolone acetonide (kenacort®) 20 mg/0.5 ml					Group II: 4
			-VAS pain >30 mm and FIHOA ≥ 6						
Bahadir et al. (2009)	Single-blind RCT	40	-Eaton stage 2 or 3	Group I: 3 weekly injections of sodium hyaluronate (ostenil®) 5 mg/0.5 ml	48	None	VAS pain (0–10); pinch strength (pound); grip strength (pound); DHI (0–90)	VAS pain decreased significantly vs baseline over 12 months in group II and over 6 months in group I. Pinch strength didn’t improve in any group, while grip strength increased significantly in both. DHI improved significantly only in group II	0
				Group II: one injection of triamcinolone acetonide (kenacort®) 20 mg/0.5 ml					
NCT00398866	Three arms RCT	200	-Unacceptable pain despite modification of activity and NSAIDs	Group I: 2 weekly injections of 1 ml of hylan GF-20 (synvisc®)	26	None	VAS pain (0–100); DASH (0–100)	Only partial results reported	Group I: 0
			-Failure/intolerance of conservative therapy with NSAIDs and/or	Group II: one injection of triamcinolone (kenalog®) 40 mg/1 ml, followed by a placebo injection of 1 ml 0.5% bupivacaine after 1 week					Group II:0
			COX-2 inhibitors	Group III: Two weekly injections of 1 ml of bupivicaine 0.5%					Group III: 1
Roux et al. (2007)	Three arms RCT	42	-VAS > 40 mm	Group I: One injection of 1 ml of sodium hyaluronate (sinovial®)	12	Yes (radioscopic control)	VAS pain (0–100); FIHOA (0–30)	No significant differences were found among the groups over the study for VAS and FIHOA. Intra-groups analyses showed significant improvement in VAS and FIHOA in group II and III, but not in group I. Efficacy was evident after 1 month and persisted at 3 months	NR
			-Failure of other therapies	Group II: 2 weekly injections of 1 ml of sodium hyaluronate (sinovial®)					
			-Kellgren grade II-IV	Group III: 3 weekly injections of 1 ml of sodium hyaluronate (sinovial®)					
Figen Ayhan and Ustün (2009)	RCT	66 joints of 33 pts	-VAS > 40 mm	Group I: One injection of 1 ml of hylan G-F 20 (synvisc®)	24	None	VAS pain (0–100); FIHOA (0–30); pinch strength (lbs)	Statistically significant improvements of VAS, FIHOA and pinch strength were observed in group I at 24 weeks, while only VAS decreased temporarily in group II at 6 weeks	NR
			-Eaton grade 1–4	Group II: one injection of 1 ml of saline solution					
Ioppolo et al. (2018)	RCT	58	-Pain duration ≥6 months	Group I: 3 weekly injections of 0.5 cm^3^ HA (sinovial mini®)	24	Yes (US guidance)	VAS pain (0–10); DHI (0–90); grip and pinch strength (kg)	A significant improvement of VAS and DHI was observed in both groups over time, but a greater average improvement was detected in group II at 24 weeks. A significant increase in strength was reported in both groups, but it was superior in group II vs group I starting immediately after the treatment	0
			-Age >40 years	Group II: 3 weekly sessions of ESWT (2,400 pulses for each session with a frequency of 4 Hz and an EFD of 0.09 mJ/mm^2^)					
			-VAS >4 mm						
			-Eaton grade 2 or 3						
Koh et al. (2019)	Retrospective comparative study	74	-Age > 40 years	Group I: One injection of 0.5 ml of sodium hyaluronate mixed with 0.5 ml of ketorolac 30 mg/ml	24	Yes (US- guidance)	DASH (0–100); VNS for pain (0–10)	The DASH and VNS scores improved at 1, 3 and 6 months in both groups, but the onset of pain relief was more rapid/at 1 month) in group I vs group II	Group I: 5
			-Failure to other conservative treatments						
			-Eaton grade 2 or 3	Group II: one injection of 0.5 ml of sodium hyaluronate mixed with 0.5 ml of saline					Group II: 0
			-Pain duration ≥3 months						
Schumacher et al. (2004)	Open-label study	16	-Pain and/or tenderness at TMCJ	5 weekly injections of sodium hyaluronate (hyalgan®) 10 mg/ml, MW 500–730 kDa	24	None	VAS pain (0–10); tenderness (0–3); crepitus (0–3); 5-question non validated hand function survey; pinch strength (kg)	Mean pain score at rest decreased of 46% and pain on use of 27% at 6 months vs baseline. No other significant improvement in the evaluated parameters were reported	2
Frizziero et al. (2014)	Open-label retrospective study	58	Not reported	3 weekly injections of 0.8 ml of HA 10 mg/ml, MW 500–730 kDa	24	None	VAS pain (0–10) at rest and on voluntary and passive movements; lateral pinch strength; morning stiffness; NSAIDs consumption (pills/days/month)	At 1, 3 and 6 months from baseline, VAS pain at rest and on movements significantly improved, as well as the duration of morning stiffness and NSAIDs consumption	15
Coaccioli et al. (2006)	Open-label study	43 (56 TMCJ in total)	-VAS spontaneous pain >40 mm	3 weekly injections of 0.5 ml of HA	7	None	VAS spontaneous pain (0–100); VAS provoked pain (0–100); grip strength (mmHg); FIHOA (0–30); NSAIDs/analgesics consumption (%)	Pain and FIHOA significantly decreased at the end of the study. A reduction of symptomatic drugs consumption was also observed	0
			-Provoked pain under pressure >60 mm						
Salini et al. (2009)	Open-label study	18	-Kellgren grade II-III	One injection of 1 ml of 0.8% HA, MW 0.8–1.2 million dalton	4	Yes (US guidance)	VAS pain at rest (0–10); VAS pain during common activities (0–10); NSAIDs consumption (nr pts and tablets/week); FIHOA (0–30); grip strength (kg); lateral and pulp pinch strength (kg)	Pain at rest and during activities significantly reduced after 1 month, as well as FIHOA. A significant decrease of NSAIDs consumption was also reported	2
			-Symptoms duration > 1 month						
Mandl et al. (2009)	Open-label study	32	-Kellgren grade II-IV	3 weekly injections of 1 ml of hylan G-F 20 (synvisc®)	26	None	VAS pain (0–100); DASH (0–100); opposition grip strength (lbs); overall pts satisfaction	VAS pain and DASH significantly improved at 26 weeks, while grip strength didn’t significantly change. VAS pain correlated with patient satisfaction at 26 weeks	4
Ingegnoli et al. (2011)	Open-label study	16 (32 TMCJ in total)	-VAS pain ≥40 mm	3 weekly injections of 0.5 ml of high MW HA (hyalubrix®)	24	Yes (US guidance)	VAS pain (0–100); FIHOA (0–30); synovial hypertrophy and PDS (0–3) assessed by US	VAS pain and FIHOA score significantly decreased after 2 weeks and are maintained at week 24. PDS significantly decrease after 2 weeks, but it was not maintained at week 24. No significant reduction of synovial hypertrophy was reported during the follow-up	0
			-Failure of prior treatments (NSAIDs, physical therapy, splinting)						
Di Sante et al. (2011)	Open-label study	31	-VAS pain ≥4 cm	3 weekly injections of 1 ml of HA	24	Yes (US guidance)	VAS pain (0–10); DHI (0–90)	A significant decrease of VAS pain was detected after 1 and 3 months, but not at 6-months follow-up. No significant differences were found for DHI at 1, 3 and 6 months	0
			-DHI ≥ 24						
Velasco et al. (2017)	Open-label study	35	-Age between 18 and 75 years	One injection of 0.7–1 ml of NASHA 20 mg/ml (durolane®)	24	Yes (fluoroscopy guidance)	VAS pain (0–10); Q-DASH (0–100); kapandji thumb opposition test (0–10); radial abduction (degrees); MCP joint flexion (degrees); strength of fist and clamp (kg); crepitus (%); morning stiffness (%); mobility difficulties (%)	Mean VAS pain decreased of 27.8% after 6 months vs baseline and a reduction >25% was already present after 1 month. All other evaluated parameters, excepted for strength of fist significantly improved at 6 months vs baseline	5
			-Eaton grade 2 or 3						
			-Pain duration at TMCJ >6 months						
			-VAS pain ≥4 cm in the target hand and <4 in the controlateral hand						
Bartoloni et al. (2019)	Open-label study	12	-VAS pain ≥40 mm	Two injections, 15 days apart, of 1 ml of hybrid HA (sinovial H-L®)	24	Yes (US guidance)	VAS pain (0–100); DASH (0–100)	VAS pain significantly decreased after 3 and 6 months- a significant improvement of DASH was reported at any evaluation times (1, 3 and 6 months)	0

^a^ All studies included patients with diagnosis of TBOA according to the ACR criteria (Altman et al., 1990).

COX-2, cycloxigenase-2; DASH, disabilities of the arm and shoulder; DHI, Duruöz hand index; EFD, energy flux density; ESWT, extracorporeal shock wave therapy; FIHOA, functional index for hand osteoarthritis; HA, hyaluronic acid; HAQ, health assessment questionnaire; MCP, metacarpophalangeal; MW, molecular weight; NASHA, nonanimal hyaluronic acid; NR, not reported; NSAIDs, non steroidal anti-inflammatory drugs; PDS, power doppler signal; PPT, Purdue Pegboard test; pts, patients; Q-DASH, quick-disabilities of the arm and shoulder; RCT, randomized controlled trial; ROM, range of motion; SF-36 PCS, short form-36 physical component summary; SF-36 MCS, short form-36 mental component summary; TMCJ, trapezio-metacarpal joint; US, ultrasound; VAS, visual analogue scale; VNS, verbal numeric scale.

Concerning the efficacy of the results, the RCTs by Stahl et al. (Stahl et al., 2005) and Fuchs et al. (Fuchs et al., 2006) showed a significant effect of both IA steroid and IA HA on pain relief (VAS) and function improvement (assessed by grip strength in the former study and by pinch grip and pulp pinch grip in the latter). However, Stahl et al. (Stahl et al., 2005) observed a significant improvement of the functional Purdue Pegboard Test (PPT), which measures the fine hand function, only in the HA group. Consistent with these results, Fuchs et al. (Fuchs et al., 2006) found a superiority of HA over steroids in all assessed parameters (VAS pain, grip power, and range of motion) in the medium-term. The more recent 6-months, single-blind, RCT by Heyworth et al. (Heyworth et al., 2008) reported no statistically significant differences among the three studied groups, of whom one was treated with two IA injections of hylan, one with a single injection of normal saline (0.9% sodium chloride) followed, after a week, by IA betamethasone, and another one with two IA injections of normal saline; however, a positive trend in hand function, assessed by Disabilities of the Arm, Shoulder, and Hand (DASH) scores, was observed in patients treated with HA. A positive trend in hand function, measured by Functional Index for Hand Osteoarthritis (FIHOA) score, was observed in patients treated with IA HA [3 weekly injections of a formulation of HA with molecular weight (MW) 500–1,000 kDa] also by Monfort et al. (Monfort et al., 2015) in a 6-months single-blinded randomized trial vs. betamethasone. These findings became particularly evident and reached statistical significance when patients with more severe symptoms (FIHOA score of at least five and VAS score of 50 or more) were considered for analysis.

These encouraging data on the HA therapy in patients with TBOA were recently confirmed by a 6-months retrospective comparative study which assessed the efficacy of a new hybrid formulation of HA vs. triamcinolone acetonide in 100 patients (Tenti et al., 2017). The Authors found both IA therapies effective in controlling pain (by VAS) and improving joint functionality (by FIHOA), but the benefits achieved were significantly superior in the HA group than in the steroid group after 1 month and until the end of follow-up. Furthermore, the HA formulation studied also resulted in an association with a significant decrease in the duration of morning stiffness and with a significant improvement of Health Assessment Questionnaire (HAQ) and physical component summary (PCS)-SF-36.

Contrasting results were reported by Bahadir et al. (Bahadir et al., 2009) in an RCT evaluating in the long-term 20 patients treated with a single injection of 20 mg triamcinolone acetonide and 20 patients who received three weekly injections of 5 mg sodium hyaluronate. Pain levels were significantly decreased in both groups, but the beneficial effect persisted until 12 months only in the steroid group; similarly, the improvement in hand functionality, assessed by the Duruoz Hand Index (DHI), reached statistical significance only in patients treated with triamcinolone.

Interestingly, the protocol of a new randomized multicenter study, the RHIZ’ART trial, aimed to analyze, for the first time, the possible synergistic effect of corticosteroids associated with HA, compared to steroid alone, in TBOA patients, was published last year (Cormier et al., 2019). The Authors would like to compare VAS pain, Cochin score, grip strength and opposition force, 3 months after a single injection of 0.5 ml of corticosteroid and 0.5 ml of physiological saline or 0.5 ml of corticosteroid and 0.5 ml of HA and would like to continue the follow-up until 12 months.

A phase three triple-blind (participants, care provider, investigator) RCT comparing the safety and effectiveness of hyaluronan (Hylan G-F20 injected once a week for two consecutive weeks) to corticosteroids (triamcinolone, 40 mg injected the first week, followed by a placebo injection of 1 ml 0.5% bupivacaine the second week) and local anesthetic (Bupivicaine 0.5% 1 ml injected once a week for 2 weeks) in relieving symptoms of TBOA has recently been completed (Clinicaltrials.gov, NCT00398866). Unfortunately, only partial results have been reported.

### Hyaluronic Acid Versus Other IA Treatment Comparators

Considering the lack of guidelines for the IA HA treatment schedule, in 2007 Roux et al. (Roux et al., 2007) compared the efficacy on pain and function of one, two, or three IA injections of 1 ml sodium hyaluronate, performed weekly under radioscopic control in the carpometacarpal joint of 44 patients. No significant differences were found among the three groups over the study period (3 months) for VAS pain and FIHOA, while intra-groups differences between baseline and the end of follow-up were significant only for patients treated with two or three injections.

In a 6-months Turkish RCT conducted in 2009, IA HA was compared to IA saline injection in 33 women with bilateral TBOA; in particular, hands of the same patient were divided to hylan G-F 20 injection and saline injection, randomly. The Authors found a significant improvement of VAS pain, FIHOA, and pinch strength at the 24^th^ week only in the hylan group, while a short-term (at the sixth week) placebo analgesic effect was described for the control group (Figen Ayhan and Ustün, 2009).

In another RCT on 58 TBOA patients, three weekly IA injections of 0.5 cm^3^ HA were compared to ESWT performed once a week for three consecutive weeks. Although a significant improvement in VAS pain, DHI score and grip and pinch strength was observed in both groups at 3 and 6 months, a greater benefit was reported in the ESWT group for all the assessed parameters (Ioppolo et al., 2018).

Finally, very recently, in a retrospective comparative study, Koh et al. (Koh et al., 2019) treated 74 TBOA patients with ultrasound-guided IA injection of 0.5 ml of sodium hyaluronate and 0.5 ml of ketorolac or 0.5 ml of sodium hyaluronate and 0.5 ml of saline. The DASH and verbal numeric scale (VNS) pain scores improved at 1, 3, and 6 months post-injection in both groups, but the pain reduction was significantly more rapid (at 1 month) after the injection of HA plus ketorolac compared to HA alone, suggesting a possible role of this combined IA therapy for a fast onset of analgesia.

### Hyaluronic Acid in Open Label Trials

In the last two decades, a variety of papers investigating the potential efficacy of different formulations of HA have been published (Table 2).

In 2004 the open-label study by Schumacher et al. (Schumacher et al., 2004) provided preliminary evidence that a cycle of five weekly injections of low MW (500–730 kDa) HA into the trapezio-metacarpal joint of 16 TBOA patients, was effective in reducing pain at 6 months follow-up, although a significant effect on pinch strength could not be observed. The beneficial effects of the same HA formulation have been subsequently confirmed by a retrospective open study conducted by Frizziero et al. (Frizziero et al., 2014). The Authors demonstrated that 58 patients treated with three weekly IA injections of low MW HA (500–730 kDa) presented a significant reduction of pain at rest and on voluntary or passive movements of flexion, extension, abduction, and rotation (on a 0–10 mm VAS scale), of morning stiffness duration and of NSAIDs consumption at any evaluation time (1, 3, and 6 months); furthermore, a significant improvement of radial and palmar ab-/adduction was registered at each follow-up visit.

The use of IA HA for TBOA was encouraged also in two different studies by Coaccioli et al. (Coaccioli et al., 2006) and Salini et al. (Salini et al., 2009); however, both trials were limited by a very short-term follow-up (1 month). In the former trial, 43 TBOA patients for a total of 56 trapezio-metacarpal joints were treated with three weekly injections of 0.5 ml HA and experienced a significant reduction of VAS pain, FIHOA score and NSAIDs/analgesic consumption, other than a significant improvement of grip strength after 1 month from the first injection (Coaccioli et al., 2006). In the latter study, a small group of TBOA patients (*n* = 18) received a single ultrasound-guided injection of a formulation of HA with a MW of 0.8–1.2 million Dalton; a significant decrease of pain at rest and during activities, as well as of FIHOA score were reported at the end of 1 month follow-up, together with a significant reduction of NSAIDs intake (Salini et al., 2009).

Other HA formulations also resulted to be beneficial for patients with TBOA in open label pilot trials. In an American study on 32 patients, a cycle of three weekly injections of hylan G-F 20 determined a significant improvement of VAS pain and DASH score (Mandl et al., 2009). In 2011, Ingegnoli et al. (Ingegnoli et al., 2011) evaluated the effects of three ultrasound-guided IA injections, performed 1 week apart, with high MW HA in 32 TB joints of 16 patients. The Authors reported a significant clinical improvement, characterized by VAS pain and FIHOA score decrease, 2 weeks after the injections and this effect persisted until 6 months. At the same time, a significant reduction of power doppler signal was observed at 2 weeks, suggesting a potential role of HA in reducing local inflammation, although this result was not maintained at week 24. In the same year, an Italian trial assessed the efficacy of an ultrasound-guided procedure for the treatment of TBOA with HA. Thirty-one patients received three weekly injections of 1 ml HA and experienced a statistically significant VAS reduction at 1 and 3 months after the end of the IA therapy, but not a 6-months follow-up; no significant differences were described for DHI at any evaluation times (Di Sante et al., 2011).

More recently, a 6-months, prospective, open-label study investigated the effects of a single IA injection of nonanimal hyaluronic acid (NASHA) into the trapezio-metacarpal joint of 35 TBOA patients. This HA formulation differs from the others above mentioned for the presence of synthetic cross-linking which creates a three-dimension gel network, responsible for an increased viscosity and half-life. The Authors reported a significant mean change from baseline in VAS pain score at any evaluation times (month 1, 3, and 6) with a reduction of 27.8% at 6 months. Further, a significant improvement of quickDASH, Kapandji thumb opposition test, radial abduction, metacarpal flexion, and strength of clamp scores were observed at the end of follow-up (Velasco et al., 2017). Finally, an open study on a small sample of patients (*n* = 12) confirmed the positive results of the above-mentioned study by Tenti et al. (Tenti et al., 2017) on the use of an hybrid formulation of HA. Indeed, the Authors reported a statistically significant reduction of VAS pain after 3 and 6 months and a significant improvement of DASH score at 1, 3, and 6 months (Bartoloni et al., 2019).

We did not report in this analysis the trial by Dauvissat et al. (Dauvissat et al., 2018) on a single injection of mannitol-modified cross-linked HA in patients with TBOA, because it evaluated only the effects on pain and not on functionality, as determined by our inclusion criteria.

### Platelet-Rich Plasma Injections

PRP is an autologous blood product derived by centrifugation of the whole blood and characterized by a high concentration of platelets above the normal levels (Marx, 2001). Many protocols for preparing PRP exist; one possibility is to include the leukocyte-containing buffy coat obtaining the so-called leukocyte-rich PRP, while another one is to exclude leukocytes resulting in the so-called leukocyte-poor PRP which is the standard PRP preparation for OA (Evans et al., 2020). Its use for the treatment of OA of large joints, particularly knee and hip OA, has emerged since the first decade of twenty-first century. The rationale of efficacy of this IA treatment lies on its ability to reverse pro-inflammatory processes and to modify the microenvironment inside the joint, restoring the articular homeostasis (Ornetti et al., 2016). In depth, after PRP injection, a subset of cytokines and growth factors, as vascular endothelial growth factor (VEGF), epidermal growth factor (EGF) insulin-like growth factor (IGF), platelet-derived growth factor (PDGF), interleukin-1 receptor antagonist (IL-1RA), soluble receptor of tumor necrosis factor-alpha (TNF-alpha) transforming growth factor-beta (TGF-beta), and many others, are released into the joint, through the degranulation of the platelets α-granules. Globally, these mediators exert an anti-catabolic and anti-inflammatory action, modulate the metabolic functions of chondrocytes and subchondral bone and stimulate fibroblasts to synthesize HA (Moussa et al., 2017).

Actually, only a very limited number of papers, often with a very small sample size and a not controlled design, investigating the possible efficacy of PRP in TBOA are published (Table 3).

**TABLE 3:** Summary of studies investigating intra-articular injections of platelet-rich plasma for the treatment of thumb-base osteoarthritis.

Authors, publication year	Study design	Sample size (pts)	Intervention	Follow-up duration (weeks)	Injection guidance	Outcomes evaluated	Results	No of reported adverse events
Loibl et al. (2016)	Open label study	10	2 injections of 1–2 ml of PRP with a platelet concentrations of 2.4 higher vs baseline, performed 4 weeks apart	24	Yes (fluoroscopic guidance)	VAS pain (0–10); DASH (0–100); mayo wrist score (0–100); grip and pinch strength (kg)	VAS significantly improved at 6 months vs baseline, as well as mayo wrist score. DASH and grip strength were unaffected. Pinch strength significantly declined at 6 months	1
Malahias et al. (2018)	RCT	33	Group I: 2 injections of 2 ml of PRP with a platelet concentrations of 2.6 higher vs baseline, performed 15 days apart	48	Yes (US guidance)	VAS pain (0–100); Q-DASH (0–100); patient satisfaction (yes/no)	After 12 months’ follow-up, PRP treatment yielded significantly better results vs steroid in terms of VAS pain, Q-DASH and patients’ satisfaction	NR
			Group II: 2 injections of 125 mg/2 ml methylprednisolone sodium succinate (solu medrol®) and lidocaine hydrochloride 2%, performed 15 days apart					
Medina-Porqueres et al. (2019)	Case report	1	3 weekly injections of 3 ml of PRP and 10% calcium chloride	48	None	VAS pain (0–10), grip and pinch strength (kg); kapandji opposition score; Q-DASH (0–100)	After 6 months, the patient reported an improvement of pain and functional disability. At 12 months, no recurrences or complications were observed	0
NCT03196310, ongoing	Three arms single-blind RCT	150 (estimated)	Group I: PRP injection	48	NR	VAS pain; DASH; pinch strength	No results posted	No results posted
			Group II: corticosteroid (kenalog) injection					
			Group III: Normal saline injection					
NCT04218591, ongoing	Double-blinded RCT	90 (estimated)	Group I: PRP injection	24	NR	VAS pain (0–10); nelson thumb score (0–100); EQ-5D (0–1); PRWHE (0–100); DASH (0–100); HADS (0–21); PCS (0–52); ROM (degrees); strength (kg)	No results posted	No results posted
			Group II: normal saline injection					

DASH, disabilities of the arm and shoulder; EQ-5D, EuroQoL-5D; NR, not reported; HADS, hospital anxiety and depression score; PCS, pain catastrophizing score; PRP, platelet-rich plasma; PRWHE, patient-rated wrist and hand evaluation; pts, patients; Q-DASH, quick-disabilities of the arm and shoulder; RCT, randomized controlled trial; ROM, range of motion; US, ultrasound; VAS, visual analogue scale.

The first one dates back to 2016 and analyzed the effect of two IA injections of 1–2 ml of PRP, administered 4 weeks apart to a small number of patients (*n* = 10). After 6 months of follow-up, the Authors reported a significant improvement of VAS pain and Mayo wrist score, while no differences vs. baseline were observed for DASH score and grip strength (Loibl et al., 2016). These results are supported by a RCT published in 2018 and assessed the efficacy of two ultrasound-guided IA PRP injections, performed 2 weeks apart, in 16 patients compared to two ultrasound-guided IA methylprednisolone and lidocaine injections at a 2-weeks interval in 17 patients. The Authors demonstrated a significant efficacy of PRP in improving pain (measured by VAS) and function (assessed by quick-DASH) both in the mid- (3 months) and long-term (12 months) with a superior effect of PRP compared to steroids at 12 months of follow-up (Malahias et al., 2018). The beneficial effect of PRP in TBOA was supported also by the case report by Medina-Porqueres et al. (Medina-Porqueres et al., 2019). The Authors reported the clinical history of a pianist affected by TBOA and treated with three weekly IA PRP injections who experienced a significant improvement of VAS pain, grip and pinch strength, and quick-DASH score after 6 months; at 12 months follow-up no recurrences or complications were identified.

There are two ongoing clinical trials with IA PRP for TBOA registered in ClinicalTrials.gov. A single-blind (patients) study with IA injections of leukocyte depleted PRP vs. triamcinolone acetonide and vs. placebo (normal saline) for TBOA started in United States in September 2018 (NCT03196310); no results have been reported yet.

Finally, a double-blind randomized trial is currently ongoing in Sweden in patients with radiological Eaton class 1–3 of TBOA comparing the efficacy of PRP vs. placebo (saline solution) (NCT04218591).

### New Emerging Intra-articular Therapies

New data are emerging about the possible use of IA injections based on mesenchymal-derived stem cell populations for the treatment of OA, due to their properties of providing mechanical support into the joint and stimulating cartilage repair and regeneration (Bosetti et al., 2016); however, the evidence for TBOA is still very limited. In Table 4 is reported the summary of the studies investigating such kind of IA treatment in TBOA.

**TABLE 4:** Summary of studies investigating intra-articular injections of mesenchymal-derived stem cell populations for the treatment of thumb-base osteoarthritis.

Authors, publication year	Study design	Sample size (pts)	Intervention	Source of MSCs	Follow-up duration (weeks)	Injection guidance	Outcomes evaluated	Main results on pain and functionality	No of reported adverse events
Centeno and Freeman (2014)	Case series	10	Group I: One injection of 0.3–1 ml of MSCs formulation in addition to a platelet product	Bone marrow (from iliac crest)	48	Yes (fluoroscopy)	VAS pain (0–10); strength (kg); ROM (degrees)	Positive outcomes were observed in pts treated with MSCs, compared with a reported worsening among the controls	0
			Group II: pts interested in the procedure, but not treated						
Bohr et al. (2015)	Case report	1	One injection of 1 ml cell-enriched lipoaspirate	Adipose tissue (from abdomen)	48	Yes (X-ray control)	Pain; DASH (0–100)	The patient reported to be free of pain after 5 weeks and reported a reduction of DASH score at 12 months	NR
Herold et al. (2017)	Open label study	50	One injection of 1 cc of lipoaspirate	Adipose tissue (from abdomen and tights)	48	Yes (radiographic control)	VAS pain (0–10); pinch strength (bar); kapandji test; DASH (0–100)	All the evaluated parameters significantly improved at all evaluation times until 48 weeks, but in patients with higher degrees of OA (eaton grade 3 or 4) the benefit was lower than in patients with eaton grade 2	5
Erne et al. (2018)	Retrospective comparative study	21	Group I: One injection of 1.3 ± 0.2 ml of autologous fat	Adipose tissue (from low abdomen)	72	None	VAS pain (0–10); DASH (0–100); grip strength (kg); pinch strength (kg); patient satisfaction (0–10)	Both treatments resulted effective in improving VAS pain and DASH without any significant differences between groups at one year follow-up; however, the time until complete symptoms resolution was significantly shorter for group I	Group I: 1
			Group II: Lundborg resection arthroplasty						Group II: 1
Haas et al. (2020)	Open label trial	89 (99 TMCJ)	One injection of 1–2 ml of autologous fat	Adipose tissue (from abdomen)	48	None	VAS pain; pinch and grip strength (kg); MHQ (0–100)	VAS pain and MHQ significantly improved from 2 to 6 weeks, respectively and continued to improve over 12 months	2
NCT03166410, ongoing	Open label study	500 (estimated)	Injection of autologous adipose-derived stromal vascular cellular fraction	NR	96	NR	Pain, function and stiffness	No results posted	No results posted
NCT04455763, ongoing	RCT	60 (estimated)	Group I: Injection of autologous adipose-derived stromal vascular cellular fraction in association with splinting	NR	24	NR	VAS pain (0–100); PRWE (0–10); global improvement; grip and pinch strength (kg); MHQ	No results posted	No results posted
			Group II: splinting alone						

DASH, disabilities of the arm and shoulder; MHQ, Michigan hand outcomes questionnaire; MSCs, mesenchymal stem cells; NR, not reported; OA, osteoarthritis; PRWE, patient-rated wrist evaluation; pts, patients; RCT, randomized controlled trial; ROM, range of motion; TMCJ, trapezio-metacarpal joint; VAS, visual analogue scale.

A case series on a small study population investigated the efficacy of fluoroscopy-guided IA injections of autologous mesenchymal stem cells, derived from bone marrow aspirate of iliac crest, administered to six patients, and compared to four participants who remained untreated. The Authors reported positive encouraging results for both pain and function after one year of follow-up, although they claimed caution for the several limitations of the study (Centeno and Freeman, 2014). Subsequently, Bohr et al. (Bohr et al., 2015) described the case of a 62-year old man, affected by TBOA, treated with cell-enriched lipoaspirate arhroplasty, after abdominal liposuction, who experienced pain relief after five weeks and a significant improvement vs. baseline of DASH score after one year. Herold et al. (Herold et al., 2017) confirmed the positive results in their prospective open study, which included 50 TBOA patients treated with IA injection of processed autologous fat. This therapy resulted beneficial in terms of VAS score, DASH score, grip, and pinch strength at 12 months follow-up. However, a sub-groups analysis showed significantly better outcomes in patients at Eaton stage 2, while only partial or no improvement in stage 3 or 4.

More recently, Erne et al. (Erne et al., 2018) performed a retrospective study aimed to compare the results of a surgical technique of trapeziectomy with autologous fat injections. Twelve patients underwent the Lundborg resection arthroplasty, while nine patients received autologous fat injection, harvested from their own abdomen. Both treatments resulted effective in improving pain and function (measured by VAS and DASH questionnaires, respectively) without any significant differences between groups at one-year follow-up; however, autologous fat injections seemed to determine a shorter time until symptoms resolution and shorter operative time compared with Lundborg arthroplasty.

Data on a wider cohort of TBOA patients (*n* = 99) treated with autologous fat injection was derived from the most recent study by Haas et al. (Haas et al., 2020). They reported that pain during activities at 2 and 6 weeks as well as 3, 6, and 12 months was significantly lower than at baseline. Furthermore, Michigan Hand Outcome Questionnaire (MHQ) scores were significantly higher at 6 weeks, 3, 6, and 12 months.

Two open label studies are currently undergoing to evaluate the safety and efficacy of injection therapy with autologous stromal vascular fraction (SVF) derived from adipose tissue (Clinicaltrials.gov, NCT03166410; NCT04455763). The SVF exerts anti-inflammatory, immunosuppressive and chondroprotective effects; due to its potential properties being tried in treating patients with different OA localizations (Pak et al., 2018).

Interestingly, still ongoing at the Cochin Hospital of Paris is an RCT aimed to evaluate the possible efficacy of IA injections of botulinum toxin A, associated with splinting, and compared to IA injection of saline associated with splinting. The rationale for use of botulinum toxin A in OA lies on its potential role in suppressing the release of some mediators involved in nociception (Gil et al., 2018).

### Safety of Intra-articular Therapy

In general, IA therapy represents a valid and safe alternative in OA patients with multiple comorbidities, for whom pharmacological treatments often present a not favorable risk/benefit ratio or are contraindicated. However, IA therapy is not free of several side effects.

In particular, corticosteroids are known to be associated with both local reactions, as skin atrophy or hypopigmentation, acute corticosteroid-microcrystalline joint flare and hemarthrosis and both systemic effects, including facial flush, hyperglycemia, blood pressure increase, Tachon’s syndrome, vagal reaction and hypersensitivity (Nguyen and Rannou, 2017). Furthermore, it is noteworthy to report the potential chondrotoxicity of IA steroids which still remains one of the more debated issues in this field. Indeed, some *in vitro* and animal studies demonstrated that corticosteroids can have an adverse effect on cartilage, especially at high doses, probably due to its ability to modulate cartilage proteins production and breakdown (Wernecke et al., 2015). From a clinical point of view, some trials showed a greater cartilage volume loss in patients treated with IA steroids compared to placebo (McAlindon et al., 2017; Zeng et al., 2019).

In the trials on TBOA patients summarized in this review, the adverse events related to IA steroids injections are not discussed in depth and rarely reported. The side effects occurred in a minority of patients and consisted mainly in temporary acute local pain starting 1–6 h after the injections and resolved spontaneously after one or two days. Only one patient reported mild skin atrophy and hypopigmentation (Day et al., 2004; Joshi, 2005; Khan et al., 2009; Jahangiri et al., 2014; Rocchi et al., 2018).

Intra-articular HA is usually recognized as a safe treatment for OA; the incidence of adverse events in RCTs, especially on knee OA, is rather low. The most frequent side effects consist of mild transient local reactions, such as pain, swelling, flares, and effusion at the site injection, while systemic events are seldom reported. Furthermore, rare cases of acute pseudoseptic reactions are observed in association with avian high MW cross-linked HA (Nguyen and Rannou, 2017). Actually, there is no evidence of a direct influence of the number of joint injections on the occurrence of side effects, while high MW and cross-linked formulations of HA were more frequently associated to local reactions and post-injection flares in comparison with intermediate or low MW (Reichenbach et al., 2007; Nguyen and Rannou, 2017).

The analysis of the literature papers on IA HA therapy for TBOA patients, confirmed what had already been demonstrated for HA treatment safety in OA in general. Indeed, several trials did not report any side effects after HA injections and others documented only minor local adverse reactions consisting of pain and/or swelling at the site injection, usually lasting a few hours and were spontaneously resolved (Schumacher et al., 2004; Stahl et al., 2005; Coaccioli et al., 2006; Fuchs et al., 2006; Roux et al., 2007; Heyworth et al., 2008; Bahadir et al., 2009; Figen Ayhan and Ustün, 2009; Salini et al., 2009; Di Sante et al., 2011; Ingegnoli et al., 2011; Frizziero et al., 2014; Monfort et al., 2015; Tenti et al., 2017; Ioppolo et al., 2018; Bartoloni et al., 2019; Koh et al., 2019). Only in two different studies evaluating high MW and cross-linked HA formulations, local adverse events of moderate intensity and needing ice, NSAIDs and/or selective cyclooxygenase-2 inhibitors (COXIBs) for resolution were recorded (Mandl et al., 2009; Velasco et al., 2017).

In 2007 Karalezli et al. (Karalezli et al., 2007) conducted a prospective study on 16 TBOA patients to analyze pain and tolerability of viscosupplementation therapy with HA. Patients underwent a cycle of three weekly injections of 0.3 cm^3^ sodium hyaluronate: eight patients under fluoroscopy control (group A) and the others without fluoroscopy control (group B). The results confirmed the tolerability of IA HA therapy, but pain and discomfort are frequent during the injection procedure with a major degree of pain experienced by subjects from group B.

Furthermore, the analysis of an American database containing data of patients with TBOA, the Truven MarketScan® Databases, revealed that both steroid both HA injections were associated with early post-operative complications after surgical treatment of TBOA. In particular, infectious complications were associated with corticosteroids injections, while wound-healing complications were found to be related mainly to IA HA therapy (Giladi et al., 2018).

The current evidence suggests a comparable safety profile of PRP to IA HA with self-limited post-injection pain and swelling representing the most frequent reported adverse events (Nguyen and Rannou, 2017). Unfortunately, there are no data available about the tolerability of PRP injections for TBOA.

The few studies on the IA therapy with mesenchymal-derived stem cell populations did not show severe complications and consisted mainly in persisting pain after the procedure injection. In particular, Herold et al. (Herold et al., 2017) observed a transient paraesthesia of branches of the superficial radial nerve that completely resolved after 2 months in two patients, while three patients underwent additional surgical treatment for insufficient pain relief induced by the injection therapy. Also, Haas et al. (Haas et al., 2020) reported that in 2% of the cases, further operation was needed for persisting pain. Similarly, Erne et al. (Erne et al., 2018) found one patient who needed revision surgery because of persisting pain.

## Discussion

The present narrative review provides an updated and comprehensive overview of the efficacy and safety of different IA injection-based therapies currently employed for the management of TBOA. Concerning IA steroids and HA, it seems that IA HA may be useful in TBOA, especially in improving functional capacity and IA corticosteroids in reducing painful symptomatology (Trellu et al., 2015; Kroon et al., 2016; Riley et al., 2019), but the current evidence remains equivocal and inconclusive. Indeed, in agreement of what has already been reported by some systematic reviews and meta-analysis with a robust methodological quality, the great heterogeneity among the trials published until now does not deserve a definite conclusion about the efficacy of these treatments and whether an injection-based therapy is more effective than another one (Trellu et al., 2015; Kroon et al., 2016; Riley et al., 2019). First of all, the studies differed for the design, with only few RCTs or retrospective comparative studies; in almost all cases, they were small single-center studies with a very limited number of patients. The population analyzed was heterogeneous, particularly for the severity of the radiological grade, evaluated according to different criteria (Kellgren-Lawrence or Eaton grade). Only twelve studies included a specific symptom threshold for inclusion (e.g., VAS ≥ 30 mm, VAS ≥ 40 mm, and FIHOA ≥ 6) (Coaccioli et al., 2006; Fuchs et al., 2006; Roux et al., 2007; Figen Ayhan and Ustün, 2009; Di Sante et al., 2011; Ingegnoli et al., 2011; Jahangiri et al., 2014; Tenti et al., 2017; Velasco et al., 2017; Ioppolo et al., 2018; Bartoloni et al., 2019). This factor is a potential source of bias in interpreting the trials’ results, considering that including participants with relatively low levels of symptoms could make less likely that a clinical meaningful difference in outcomes could be obtained. For these reasons, both Osteoarthritis Research Society International (OARSI) and European Society for Clinical and Economic Aspects of Osteoporosis, Osteoarthritis and Musculoskeletal Diseases (ESCEO) recommendations for the conduct of pharmacological clinical trials in hand OA recommend a minimum cut-off for inclusion in terms of pain and function (Kloppenburg et al., 2015a; Reginster et al., 2018).

Another important source of heterogeneity is represented by different formulations of IA corticosteroids and HA tested with different injected volumes. Among steroids, triamcinolone acetonide, methylprednisolone and betamethasone are the most frequent used; there are no evidence supporting the superiority of a formulation over another one in TBOA, although in large joints OA, triamcinolone acetonide seems to have a greater effectiveness (Cushman et al., 2018). The HA preparations explored in the above discussed trials included HA of different MW (low, intermediate, and high), hylan, cross-linked HA and hybrid formulations. Unfortunately, no data are available about a possible difference in efficacy according to MW and viscosity in TBOA. The number of injections was variable ranging from one to three injections both for steroids and HA, as well as the technique of IA injections. In this sense, particularly debated was the accuracy of TBOA injections with and without imaging guidance, nowadays represented essentially by ultrasound. Indeed, the consensus statement on viscosupplementation (Henrotin et al., 2015) suggested to inject the trapezio-metacarpal joint under fluoroscopy or ultrasonography guidance and a recent United States cadaveric study showed a 25% higher accuracy when thumb-base joint was injected with ultrasound guidance compared to no imaging control (To et al., 2017). Conversely, other studies demonstrated success rates comparable with those obtained under ultrasound-control when the injections were performed by an experienced physician based on palpation of landmarks (Helm et al., 2003; Mandl et al., 2006).

Furthermore, another important element of heterogeneity is represented by a great variety of analyzed outcomes. Outcome Measures in Rheumatology (OMERACT) consensus recommended to evaluate in hand OA clinical trials pain, functional capacity, joint activity, and patient global assessment (Kloppenburg et al., 2015b). Few studies followed these suggestions, and for hand functionality different scores were often used, sometimes evaluating not only the hand, but the arm in its globality; few papers investigated FIHOA, validated in hand OA and considered a reliable measure of hand functionality (Kloppenburg et al., 2015b).

Also, the times of follow-up are extremely variable, ranging from 1 to 12 months, contributing to make difficult the comparison across the studies.

Another important point often poorly explored is represented by the description of the concomitant pharmacological and non-pharmacological therapy for TBOA. Indeed, in real-world application, TBOA is managed not only with injections, but with a multidisciplinary approach, so we think that more detailed information, particularly on the concomitant use of NSAIDs/analgesics and splint, can provide useful clinical implications.

Concerning PRP and mesenchymal-derived stem cell populations injections, the data are encouraging, but still too limited for any kind of conclusion. In particular, the small sample size of the analyzed studies makes it very difficult to extrapolate the results to a large scale population. Furthermore, a better understanding of the mechanism of actions of PRP and mesenchymal-derived stem cell populations and a standardized preparation method are needed to achieve a higher level of evidence in this field. It is possible that in the future both therapies can obtain a place in the management of TBOA, mainly thanks to their properties of promoting healing cartilage defects, stem cell proliferation and preventing chondrocytes and extra-cellular matrix degradation (Bonetti et al., 2020).

The tolerability of all the discussed IA therapies were found to be quite good. Local side effects are the most frequently reported and consisted mainly of painful, moderate, local inflammatory reactions at the injection site. Corticosteroids injections have the disadvantages to potentially determine skin and/or ligaments alterations, particularly in the case of repeated injections and in diabetic subjects. However, the most serious risk for IA injections remains septic arthritis which has not been described in any of the above-presented studies.

The current review of the scientific literature allowed us to find out some important points which, in our opinion, deserve further investigation. First of all, the discrepancy between the clinical experience of several physicians with expertize in this field, and the published recommendations from international scientific societies has become more evident throughout the last few years. This gap deriving from the literature evidence, which is methodologically very poor, is likely to determine negative implications, restricting patients’ access to this valuable treatment option and accelerating the referrals to the surgery, a more expensive strategy and without minor risks. In our opinion, the only way to solve this discrepancy is to realize well-designed and well-conducted controlled trials, preferably double-blind RCTs or real-life studies on a large sample size of patients. Further, there is a need for homogenous trials which can follow the OARSI and ESCEO criteria for the conduct of clinical studies in hand OA, not only in selecting patients, but also in defining the most reliable pain and function outcomes (Kloppenburg et al., 2015a; Reginster et al., 2018). The follow-up should be performed in the long-term with results at 1 year. The injection procedure should be standardized, as well as the schedule of the injected agent. At this regard, we think that studies of comparisons between the different IA therapies and placebo, between different agents within the same class and between different IA treatment belonging to various pharmaceutical categories should be encouraged. Also comparing the injection-based therapies with other conservative strategies including oral pharmacological drugs, exercise, splint, different kinds of physical therapies, as laser therapy or extracorporeal shockwave therapy, should be very interesting. Finally, to understand if some disease characteristics (e.g., radiological grade) could be useful in helping clinicians in the choice of the IA therapy, should be desirable.

The main limitation of this review lies in its narrative nature with all the limitations inherent to a non-rigorous systematic review. In particular, this paper did not identify the quality and the strength of the discussed trials, and has not been built on a robust methodology structure. Further limitations are those intrinsic to the included papers which presented several consistent methodological flaws, as the not randomized controlled design.

## Conclusion

The intra-articular injection of therapeutic agents is an attractive strategy for the local treatment of TBOA, which takes a place within the multidisciplinary approach for the management of hand OA. However, the current evidence remains equivocal. The main reason behind this is related to the poor methodology of the available scientific studies, which makes the results quite inconclusive. Some data supported the clinical usefulness of IA HA, especially in improving functional capacity and of IA corticosteroids in reducing painful symptomatology; new emerging and encouraging results derived from PRP and mesenchymal-derived stem cell populations, but they are still preliminary. At this regard, we auspicate a growing development of the scientific evidence in the field of regenerative medicine until now poorly explored in TBOA. For an exhaustive understanding of all therapeutic possibilities related to the different intra-articular agents in TBOA patients there is a need for large, independent, methodologically robust RCTs with long-term follow-up.

## Research Agenda

• To publish well-conducted double-blind RCTs on a large TBOA population and with a long-term follow-up
• To use standardized selection criteria and standardized efficacy outcomes to make the different studies uniform and comparable
• To uniform the injection technique and the therapeutic regimens (dosage, number of injections, kind of formulation of steroid and HA)
• To compare the IA agents with each other, with placebo, and with other conservative therapeutic options
To find out if a corticosteroid or HA formulation is superior to another one in TBOA
• To study the additional symptomatic effect of the different IA therapies, combined with other therapeutic options such as pharmacological management, physiotherapy and splinting
• To identify patients and disease characteristics useful to guide the choice of the IA agent
